# Grooming and etiquette as part of nurse's professionalism: An essential curricular competency

**DOI:** 10.12669/pjms.35.2.647

**Published:** 2019

**Authors:** Sachal Aqeel Safdar, Lubna Aqeel

**Affiliations:** 1 *Sachal Aqeel Safdar, MBBS (Final Year), Shifa College of Medicine, Shifa Tameer e Millat University, Islamabad, Pakistan*; 2 *Lubna Aqeel, BSc (Hom Econ), College of Nursing, Armed Forces Postgraduate Medical Institute (AFPGMI), National University of Medical Sciences (NUMS), Rawalpindi, Pakistan*

**Keywords:** Education, Grooming, Medical etiquette, Nurses

## Abstract

**Objective:**

The B.Sc. Nursing has limited hours dedicated to grooming and personal development. A smartly turned out, trained empathetic and efficient nurse helps in alleviating the miseries of the patients. The objective of this study was to see the acceptability and perceived usefulness of a course on grooming and etiquette.

**Methods:**

A proforma based qualitative study was carried out at College of Nursing, AFPGMI & National University of Medical Sciences (NUMS), Rawalpindi, from Jan to Dec, 2017. An eight week course, with weekly lectures was conducted for first to fourth year classes. At the end a semi-structured proforma with simple open ended questions was administered. The questions were both in English and Urdu. The results were analyzed by finding percentages of positive and negative responses. The descriptive responses were grouped in recurring themes and analyzed for content and their constructive value.

**Results:**

There were 186 nursing cadets who completed the course and filled the proforma (55, 52, 45 and 34 from first year to fourth year, respectively). Out of 186, 98.9% (184) thought that this was a useful course. All topics were considered useful. Most liked were communication skills, teaching visits to wards, hostels and balancing home and work. About 7% disagreed about the strict dress codes, but agreed with the proper demeanor. Many suggested that senior nurses also have a dire need of these grooming lectures as many used derogatory language. Also pointed out was absence of a mechanism to report a lapse in ethical conduct seen in a colleague or a senior.

**Conclusion:**

Nursing is a demanding profession. It is important that our training includes sensitization to the non-technical attributes. They have to be groomed to become role models for young talented girls to join this noble profession. Even a short course may help to sensitize these young ladies to a very important aspect of their lives and profession.

## INTRODUCTION

Patients who come to our hospitals are not in their best of health or mood. Most of the times, in Emergency Rooms or Wards, Nursing Officers are the main Health Professionals they come in contact first. They are the face of our health service and hospitals.^1^ A smartly turned out, appropriately trained, efficient and empathetic Nurse goes a long way in alleviating the miseries of our patients. If she does not have the appropriate standard of manners, the chances are that they would be even more put off. This mismatch can bring the whole experience to be less than ideal.

The primary goal should be to project a positive image. The non-verbal messages are as important as the verbal skills. Like the ABC of Basic Life Support, the ABC of grooming consists of “Appearance, Behaviour, Communication”.^1^,^2^ Dedicated grooming sessions early in their training can launch the new impressionable student nurses on a course which would be standardized and beneficial not only for the nurses but also for the service and patients.

These expectations are not only of medical competence but a truly caring and concerned attitude. Most of the complaints generated in the hospital regarding nursing care are not of medical nature but usually about the attitudes and behaviours.^3^ Nursing is not only learning the basics of profession but maintenance of confident posture, dress code and the way they conduct themselves, even under stressful situations work rewards encourage nurses to adopt a more proactive attitude toward work-related adversity.^4^ It is important for the nurse to pay attention to the aesthetics of own grooming and exude a class, embedded in the professionalism of nursing.^5^

The curriculum of the four year BSc Nursing degree has limited hours dedicated to the grooming and personal development.^6^ No one is a born professional. It is learned through school teaching, work experience, and the role-modeling of more senior staff. Moreover, if professionalism is a set of behaviors, then it can be measured.^7^ There are neither structured competencies included, nor any objective methods of evaluation. A dedicated and focused course of 8 weeks, with lectures, interactive discussions and hands-on practice was conducted for all the four classes from 1^st^ to 4^th^ year. It was envisaged that it may help to bring these young bright girls from diverse backgrounds to learn how to conduct themselves gracefully while taking care of sick. This would not only enhance their self-esteem, but bring this noble profession at par with the most respected and dedicated professions of the country.

The objective of this study was to see the acceptability and perceived usefulness of this eight weeks course on grooming and etiquette through a simple feedback proforma administered at the end of the eight weeks course to all the four classes of BSc Nursing.

## METHODS

This was a simple Proforma based Qualitative study. It was conducted at College of Nursing, Armed Forces Postgraduate Medical Institute (AFPGMI) & National University of Medical Sciences (NUMS), Rawalpindi, Pakistan over a period of 12 months from January to December 2017. Approval was obtained from the Institutional Review Board.

A short course of eight weeks, with one hour long lecture per week was conducted for first to fourth year classes of nursing cadets of College of Nursing. All the classes were conducted by one of the authors (LA) over a period of one year. An outline of topics and expected competencies in this short initial program is given in Table-I.

**Table I:** Curriculum of Course of Grooming Classes of 8 weeks.

Weeks	Topics	Objectives	Venue & Method
Week 1	Orientation	- Introduce self and the cadets - Discuss the significance of their profession - Discuss why it is important to have manners and grooming - Outline of the eight weeks and what topics are to be covered	-Lecture Hall -Interactive, friendly & enthusiastic
Week 2	Communication Skills 1 With doctors, colleagues and other staff members.	- Different ways of talking to the doctors, colleagues and other lower staff - Importance of being able to convey your point, decently and effectively - Importance of carrying out duties diligently & helping colleagues - Ensuring not to degrade juniors and being supportive - Lead by example - Learning from good seniors and ensuring not to pick up bad habits from rude colleagues and seniors	-Lecture Hall -Interactive, friendly & enthusiastic -Use examples from daily life, ask their experiences, share your own
Week 3	Communication Skills 2 With patients and their families.	- Importance of having good communication with patients and their families - How to deal with rowdy and disruptive patients - How to follow and deliver instructions and information - How to deliver bad news	-Lecture Hall -Interactive, friendly & enthusiastic -Use examples from daily life Role Play & Videos
Week 4	Presenting yourself at work place, social events and during visits	- Importance of good manners - Appropriate dress - Conducting yourself gracefully - Giving respect to all Seniors - Importance of Protocol and Respect	As Above
Week 5,6	Learning etiquettes in the Hostels, Mess and Wards, (with special reference to Armed Forces Culture).	- Importance of this session - Table setting - Serving - Eating manners - Etiquettes	This session will be conducted in the Wards and Nurses’ Hostel’s Mess, with the class
Week 7	How to balance home and work.	- Importance of balance - Not to bring problems of ‘work’ in ‘home’ and vice versa - How to deal with problems - What not to say or do at home to ensure harmony with own family	-Lecture Hall -Interactive, friendly & enthusiastic -Use examples from daily life Role Play
Week 8	Revision, Evaluation and Feedback.	- Revision of all the sessions - Lesson learnt - Getting written feedback from cadets - Discussing any suggestions	-Lecture Hall -Interactive -Simple proforma (sample in Annex A)

No formal written or oral test was conducted. At the end of the eight weeks course, an interactive session was held about what was learned and revised. A semi-structured proforma with simple open ended questions was administered (Annex A). The questions were in English and Urdu, to ensure they are understood by the cadets and they can freely answer them in a frank and free manner. It was anonymously filled. The results of the proforma were analyzed by finding percentages of positive and negative responses. The free hand descriptive responses were grouped in recurring themes and analyzed for content and their constructive value. Frivolous responses and personal problems were identified but not included in the evaluation reports, although they were shared with the class in-charge tutors to determine their nature and address appropriately, ensuring that their genuine grievances are heard. A written summary was shared with the Director of Nursing in the Medical Directorate, the highest body over-seeing nurse’s training, with a view to discuss at the Nursing Council meetings.

## RESULTS

There were 186 nursing cadets and all those who completed the course were instructed to fill the proforma (55, 52, 45 and 34 from first year to fourth year, respectively). The response was overwhelming. Out of 186, 98.9% (184) thought that this was a useful course.

There were three simple questions in the feedback. The first was about what they learned from this course had the following main themes:

- They highlighted each and every topic and felt they were useful.
- In order of liking they felt that all were important, but communication skills, visits and learning during teaching visits in the wards and hostels and balancing home and work for a nurse were important soul searching topics.
- About 7% disagreed about the strict dress and presentation codes discussed, but overall agreed with the proper demeanor.
- They felt it was a nice change from the monotonous academic lectures
- The senior nurses in the wards also have a dire need of these grooming lectures. Many thought that they use excessively derogatory language, which is un-becoming of a senior officer. These lectures can provide a sensitization to them.

To the second question about what was not useful in this program, they all agreed that all topics were useful and should be kept as such. The third question which was most important, about how to improve the curriculum of future courses, the main responses were:

- This or similar course should be part of the BScN course and taught as an integral part of their training.
- The role plays need to be increased as it is more enjoyable and conveys the message in a more forceful manner
- If a lapse in ethical conduct is seen in a colleague or a senior, there should be a mechanism to report it and addressed.
- Many suggested an increase in time, and instead of only eight weeks, there should be class every week throughout their year, every year.

Then there were some comments and suggestions not related to the course but to the residential and work environment, which were noted and conveyed to the appropriate authorities.

## DISCUSSION

A focused program, consisting of interactive lectures, role plays and on-job sensitization experiences can be incorporated in the different years of the four year BScN course. There have been fundamental changes in the way our nursing staff has looked after our patients. The newer nurses are more educated, computer literate and more focused, which has been found to be useful.^8^ The number of patients has increased and they are also more educated and discerning. Even the families of patients from under-developed areas are more aware. They have access to internet and cable TV and are more connected. The patients’ expectations from our nursing staff are of the highest level of professionalism.

These expectations are not only of medical competence but a truly caring and concerned attitude, highlighting the multidimensionality of the concept of competence.^9^ Most of the complaints generated in the hospitals regarding nursing care are not of medical nature but usually about the attitudes and behaviours. Nursing is not only learning the basics of profession but maintenance of confident posture, dress code and the way they conduct themselves, even under stressful situations. According to one study the senior matrons reported that they preferred a ‘good, kind and reliable’ nurse over a ‘clever’ nurse.^10^ There are wide reaching implications for positive nurses’ attitude and improved ethics education that may motivate ethical conduct throughout student nurses’ careers. This grooming of ethical mind-set can be motivated by focused programs during their educational cycle.^11^ There is even more awareness in the ever growing private sector in Pakistan, where the grooming aspect is emphasized much more than public sector.^12^ Appropriate ethical training methods and good role models can help students acquire attributes that are of utmost importance for the nursing profession and combined with the other attributes they already have can produce a fulfilling experience for the young nurse.^13^

During the feedback to the third question many students pointed out the rude behavior of the senior nurses in the wards, which amounted to bullying. This was more from their own kind, and not as much from other health professionals or patients. In seeking remedies to the problem of workplace bullying, nurse leaders need to focus on why this occurs and on ways to reduce it. This is a critical issue, since it is linked with nurse attrition and eventually to their morale and self-efficacy.^14^,^15^ The victims of bullying are subjected to being terrorized, annoyed, excluded, belittled, deprived of resources, isolated and prevented from claiming rights. The young nursing cadets who experience bullying have decreased job satisfaction, work performance, motivation and productivity. Bullying also negatively affects victims’ social relationships inside and outside the institution.^16^

A major component of the program addressed the appearance, uniforms and general presentation of the nurses. Almost all agreed in the feedback that it was an important aspect which affected the patients’ response and behavior during the first interaction with the nursing staff. Findings are inconsistent but demonstrate that a standardized uniform style neatly turned out nurse increased the perception of professionalism and recognition of nurses among patients.^17^ One study examined nurse professionalism by assessing the nurse image traits of eight uniforms of different patterns, colors and styles, as perceived by pediatric patients, adult patients, and adult visitors. Traits of nurse professionalism were highest in white uniforms. Future research is needed in our country to determine if this applies to our society.^18^ According to Verma, personal grooming is just as important as what is worn. Neglecting personal hygiene can ruin the image you wish to present. Clean hair, trimmed, and neatly combed; neat fingernails clean, and trimmed; teeth Brushed and fresh breath; sparingly used make-up; just enough perfume as not to linger after you leave; these are some age old advice given by Verma.^1^

Another major part of this program focused on communication skills of the nurses. This includes communication not only with the patients and their relatives, but also with juniors and senior health professionals. The feedback also emphasized the continued inclusion of this aspect in the Grooming program, especially enjoying the role plays during the sessions. This aspect has been highlighted in nursing training institutes all over the world with many schools and hospitals taking steps to ensure improvements. Good communication is central to quality care, yet nurses often concede that it is not always effective with patients and not enough emphasis is given in the curriculum.^19^

Etiquette is more than good manners; it’s a tool for cultivating good relationships. More than most careers, nursing are characterized by professional relationships among different people in numerous settings.^20^ Nursing etiquette and grooming are old-fashioned terms, but many of the social graces first described are still relevant today so that we may continue to promote a competent and professional image.^21^

The four key professional attributes, autonomy, advocacy, accountability and assertiveness, along with the virtues listed in the themes above, exemplify the ‘good’ nurse and are identified as the linchpins of modern professional ethics and good conduct.^22^

### Limitations of the study

The study was conducted in one nursing college and therefore, the findings can only be used to specific participant group where the study was conducted.

## CONCLUSION

Nursing is a demanding profession. It is important that our training includes sensitization to the non-technical attributes of a nurse. She has to be groomed to become a person who inspires other young talented girls to join this noble profession; to command respect in the society; to inspire patients and their relatives to get better; to become an epitome of goodness. Lectures are perhaps not the only way to achieve this. Role models have to be propagated and projected. But these few interactive hours may help to sensitize these young ladies to a very important aspect of their lives and profession.

### Author’s Contribution

**SAS** conducted, designed, analyzed the data, wrote and revised the manuscript.

**LA** conceived, collected the data and reviewed the manuscript.

## Appendix

**ANNEX-A:** ***Question-1:*** Were these lectures useful and if yes what did you learn from these lectures? ***Question-2:*** What was not useful in these lectures? ***Question-3:*** How can we improve these lectures in the future?
